# Divergent Responses of Different Endothelial Cell Types to Infection with *Candida albicans* and *Staphylococcus aureus*

**DOI:** 10.1371/journal.pone.0039633

**Published:** 2012-06-22

**Authors:** Kati Seidl, Norma V. Solis, Arnold S. Bayer, Wessam Abdel Hady, Steven Ellison, Meredith C. Klashman, Yan Q. Xiong, Scott G. Filler

**Affiliations:** 1 Los Angeles Biomedical Research Institute at Harbor-UCLA Medical Center, Torrance, California, United States of America; 2 University Hospital Zurich, University of Zurich, Zurich, Switzerland; 3 David Geffen School of Medicine at UCLA, Los Angeles, California, United States of America; 4 Department of Biology, California State University-Dominguez Hills, Carson, California, United States of America; University of Aberdeen, United Kingdom

## Abstract

Endothelial cells are important in the pathogenesis of bloodstream infections caused by *Candida albicans* and *Staphylococcus aureus*. Numerous investigations have used human umbilical vein endothelial cells (HUVECs) to study microbial-endothelial cell interactions *in vitro*. However, the use of HUVECs requires a constant supply of umbilical cords, and there are significant donor-to-donor variations in these endothelial cells. The use of an immortalized endothelial cell line would obviate such difficulties. One candidate in this regard is HMEC-1, an immortalized human dermal microvascular endothelial cell line. To determine if HMEC-1 cells are suitable for studying the interactions of *C. albicans* and *S. aureus* with endothelial cells *in vitro*, we compared the interactions of these organisms with HMEC-1 cells and HUVECs. We found that wild-type *C. albicans* had significantly reduced adherence to and invasion of HMEC-1 cells as compared to HUVECs. Although wild-type *S. aureus* adhered to and invaded HMEC-1 cells similarly to HUVECs, an *agr* mutant strain had significantly reduced invasion of HMEC-1 cells, but not HUVECs. Furthermore, HMEC-1 cells were less susceptible to damage induced by *C. albicans*, but more susceptible to damage caused by *S. aureus.* In addition, HMEC-1 cells secreted very little IL-8 in response to infection with either organism, whereas infection of HUVECs induced substantial IL-8 secretion. This weak IL-8 response was likely due to the anatomic site from which HMEC-1 cells were obtained because infection of primary human dermal microvascular endothelial cells with *C. albicans* and *S. aureus* also induced little increase in IL-8 production above basal levels. Thus, *C. albicans* and *S. aureus* interact with HMEC-1 cells in a substantially different manner than with HUVECs, and data obtained with one type of endothelial cell cannot necessarily be extrapolated to other types.

## Introduction

Endothelial cells play a crucial role in the pathogenesis of many types of human infections [1], [2]. For example, after a microbial pathogen enters the circulation, it must adhere to and invade the endothelial cell lining of the blood vessels to infect deeper tissues to cause organ dissemination. In addition, by expressing pro-inflammatory cytokines and leukocyte adhesion molecules, endothelial cells recruit phagocytes to foci of infection and are therefore essential for orchestrating the host defense against microbial pathogens.

Because of the importance of endothelial cells in the pathogenesis of bloodstream infections, numerous investigators have used *in vitro* models of microbial-endothelial cell interactions to study the mechanisms by which distinct microbial pathogens adhere to, invade, damage, and activate endothelial cells. Many of these investigations have used human umbilical vein endothelial cells (HUVECs) [3]–[15]. For example, mutants of *Candida albicans* with reduced capacity to damage HUVECs *in vitro* are likely to have attenuated virulence in a murine model of hematogenously disseminated candidiasis [13]. Also, the capacity of clinical isolates of *Staphylococcus aureus* to damage HUVECs is directly correlated with their virulence in the rabbit model of infective endocarditis, and inversely correlated with their response to vancomycin in this animal model [14]. Thus, these investigations demonstrate that HUVECs may serve as a useful *in vitro* model of host-pathogen interaction.

There are some disadvantages to using HUVECs for such studies. Firstly, because they are primary cells, they exhibit significant donor-to-donor variability in some microbial interactions [16]. Secondly, they have a relatively short life span *in vitro*, and their phenotype can change with successive passages. Thirdly, HUVECs can be difficult to transfect, and their finite life span makes it problematic to develop stably transfected cell lines. Finally, the availability of HUVECs may be constrained by medical and ethical issues.

To overcome these problems, immortalized endothelial cell lines have been developed. These cell lines have the advantages of easier maintenance, longer life span, less variability, and better availability. However, immortalization may lead to functional defects, such as altered expression of leukocyte adhesion molecules [17], [18]. In addition, endothelial cells from different vascular beds have a diversity of phenotypes in terms of their cell morphology, function, gene expression, and antigen composition (Reviewed in [19], [20]). Thus, endothelial cells from different anatomic sites may exhibit marked differences in their interactions with microbial pathogens.

One immortalized cell line that has been used in studies of microbial pathogenicity is the HMEC-1 cell line. This cell line was developed by transfecting dermal human microvascular endothelial cells from human foreskin with a plasmid containing the simian virus 40A gene [21]. These cells have been used to study the endothelial cell interactions of multiple microorganisms, including *Chlamydia pneumoniae* [22], *Brucella* spp. [23], *Bartonella henselae* [24], *Mycobacterium tuberculosis* [25], *Orientia (Rickettsia) tsutsugamushi* [26], *Rickettsia rickettsii* [27], *Candida albicans* [28], [29], and *Staphylococcus aureus* [30].

In order to evaluate the usefulness of HMEC-1 cells for studying different aspects of endovascular infection, we compared the interactions of *C. albicans* and *S. aureus* with these cells and HUVECs. We discovered that *C. albicans* and *S. aureus* interacted with HMEC-1 cells in a significantly different manner as compared to HUVECs.

## Results

### *C. albicans*, but Not *S. aureus,* has Reduced Adherence to and Invasion of HMEC-1 Cells

**Figure 1:**
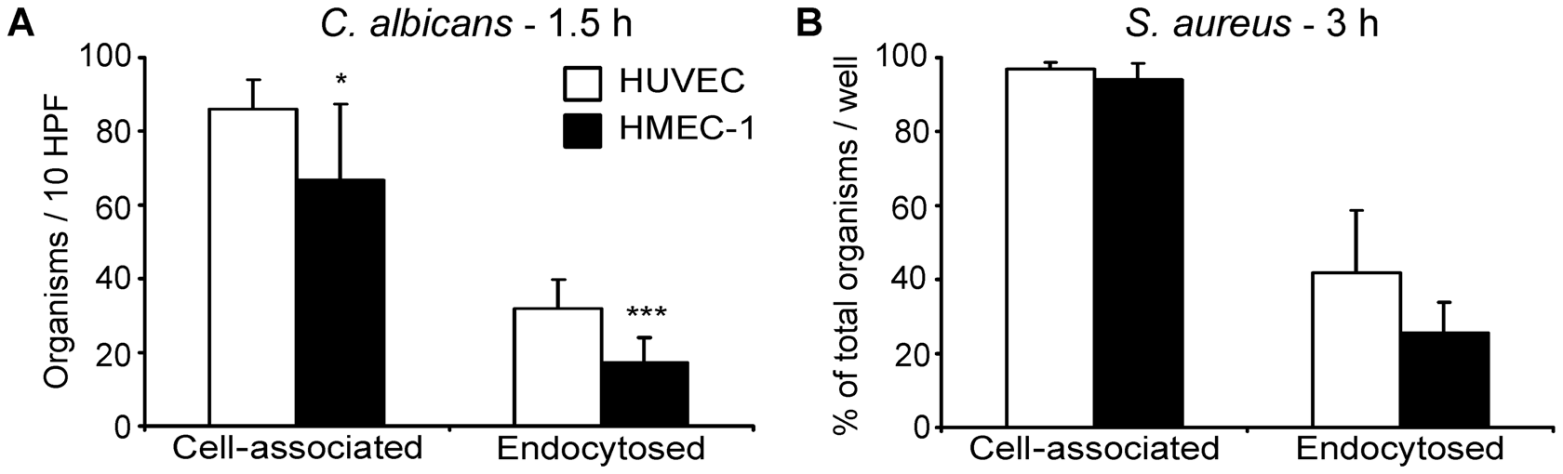
Adherence to and invasion of human umbilical vein endothelial cells (HUVECs) vs. HMEC-1 cells by wild-type *C. albicans* and *S. aureus*. Adherence and endocytosis of *C. albicans* strain CAI4+CIp10 was assessed 1.5 h after infection at a MOI of 1 (A). Adherence and endocytosis of *S. aureus* strain 6850 was determined 3 h after infection at a MOI of 1 (B). *, *p*<0.05 and ***, *p*<0.001 in HMEC-1 vs. HUVECs.

We first compared the capacity of *C. albicans* and *S. aureus* to adhere to and invade HMEC-1 cells and HUVECs. Because *C. albicans* invades and damages endothelial cells much more rapidly than does *S. aureus* [6], [9], [10], [12], [14], [15], [31], [32], the *C. albicans*-endothelial cell interactions were assessed at earlier time points than the *S. aureus*-endothelial cell interactions. We found that wild-type *C. albicans* cells had 23% lower adherence and 47% less invasion of HMEC-1 cells compared to HUVECs (*p*<0.05 and *p*<0.001 for adherence and invasion, respectively) (**Fig. 1A**). In contrast, a wild-type strain of *S. aureus* adhered to and invaded HMEC-1 cells similarly to HUVECs (**Fig. 1B**).

Next, we investigated whether microbial adherence to and invasion of HMEC-1 cells and HUVECs occur via the same mechanism(s). *C. albicans* Ssa1 and Als3 are invasin proteins that are necessary for maximal endothelial cell adherence and invasion (**Table 1**, [12], [15]). We found that *ssa1*Δ/Δ and *als3*Δ/Δ mutant strains were defective in their capacity to adhere to and invade both HMEC-1 cells and HUVECs (**Table 2**). The magnitude of these defects was similar for both HUVECs and HMEC-1 cells, indicating that Ssa1 and Als3 mediate adherence to and invasion of both types of endothelial cells.

**Table 1:** *C. albicans* and *S. aureus* strains used in this study.

Strain	Description	Reference
*C. albicans* strains		
DAY185	*ura3*Δ*::λimm434 ARG4::URA3::arg4::hisG his1::hisG::pHIS1* *ura3*Δ*::λimm434 arg4::hisG his1::hisG*	[54]
CAI4+CIp10	*URA3::ura3*Δ*::λimm434 rps10*Δ*::pCIP10* *ura3*Δ*::λimm434 RPS10*	[55]
*ssa1*Δ*/*Δ*-URA3*	*ura3*Δ*::λimm434 ssa1::FRT ssa2::FRT rps10::URA3* *ura3*Δ*::λimm434 ssa1::FRT SSA2 RPS10*	[15]
*ssa1*Δ/*SSA1*	*ura3*Δ*::λimm434 ssa1::FRT ssa2::FRT rps10::URA3::SSA1* *ura3*Δ*::λimm434 ssa1::FRT SSA2 RPS10*	[15]
CAYF178U	*ura3*Δ*::λimm434::URA3-IRO1 als3::ARG4 arg4::hisG his1::hisG* *ura3*Δ*::λimm434 als3::HIS1 arg4::hisG his1::hisG*	[12]
CAQTP178U	*ura3*Δ*::λimm434::URA3-IRO1 als3::ARG4::ALS3 arg4::hisG his1::hisG* *ura3*Δ*::λimm434 als3::HIS1 arg4::hisG his1::hisG*	[12]
*S. aureus* strains		
6850	Wild type clinical osteomyelitis isolate, MSSA	[33]
JB-1	Menadione auxotroph SCV from strain 6850	[33]
300-169	Clinical blood MRSA isolate, *agr*-I, SCC*mec* IV, CC45	[35]
300-169Δ*agr*	300-169 *agr::tet*(M), Tc^r^	[35]

Tc^r^, tetracycline-resistant.

**Table 2:** Interactions of different *C. albicans* and *S. aureus* mutants with HUVECs and HMEC-1 cells^a^.

		HUVEC	HMEC-1	*p*
Strain	Interaction	(% ± SD of corresponding parental strain)		(HUVEC vs. HMEC-1)
*C. albicans* b				
*ssa1*Δ*/*Δ	Adherence	66.4±23.1**	71.3±29.2*	0.697
	Endocytosis	45.3±29.7***	44.6±27.0**	0.183
*als3*Δ*/*Δ	Adherence	52.6±9.5***	44.9±18.6***	0.288
	Endocytosis	4.0±2.6***	2.6±1.1***	0.958
*S. aureus* c				
6850 JB-1 (SCV)	Adherence	88.3±5.2**	96.2±5.7	<0.05
	Endocytosis	212.5±96.5*	202.6±99.5	0.887
300-169*agr*Δ	Adherence	99.8±1.4	100.2±0.7	0.887
	Endocytosis	92.0±17.3	68.5±17.9**	0.061

a , Data are expressed as % of the corresponding parental strain (set as 100%).

b , *C. albicans* binding was tested after 1.5 h at an MOI of 1.

c , *S. aureus* binding was tested after 3 h at an MOI of 1 and expressed as % of the total number of bacteria per well.

* , *p*<0.05; **, *p*<0.01 and ***, *p*<0.001 vs. corresponding parental strain. Endothelial cell adherence/endocytosis of parental strain was set as 100%.

We also evaluated the adherence and invasion of two *S. aureus* mutants. One strain was JB-1, a stable gentamicin-induced small-colony variant (SCV) of the clinical parental strain, 6850 (**Table 1**). SCV strains are known to persist within endothelial cells, while causing little damage [14], [33], [34]. The second strain was an *agr* deletion mutant of clinical MRSA isolate 300-169 (**Table 1**, [14], [35]). *agr*, the accessory gene regulatory locus of *S. aureus* governs the expression of many adhesins and secreted virulence factors, such as proteases and toxins, and is known to affect host cell binding and invasion [14], [32], [34]. We found that while the SCV mutant adhered to HMEC-1 cells similarly to its wild-type parent strain, it had slightly decreased adherence to HUVECs (**Table 2**). Also the SCV strain had increased capacity to invade both types of endothelial cells as compared to parental strain, 6850 (**Table 2**). Although the adherence of the *agr* mutant to HMEC-1 cells and HUVECs was similar to that of its wild-type parental strain, this mutant was defective in invading HMEC-1 cells, but not HUVECs (**Table 2**). There was a non-significant trend (*p* = 0.06) towards reduced invasion of HMEC-1 cells compared to HUVECs by the *agr* mutant, suggesting that *agr* may be required for *S. aureus* to maximally invade HMEC-1 cells, but not HUVECs.

### HMEC-1 Cells and HUVECs Differ in their Susceptibility to Damage Caused by *C. albicans* and *S. aureus*

**Figure 2:**
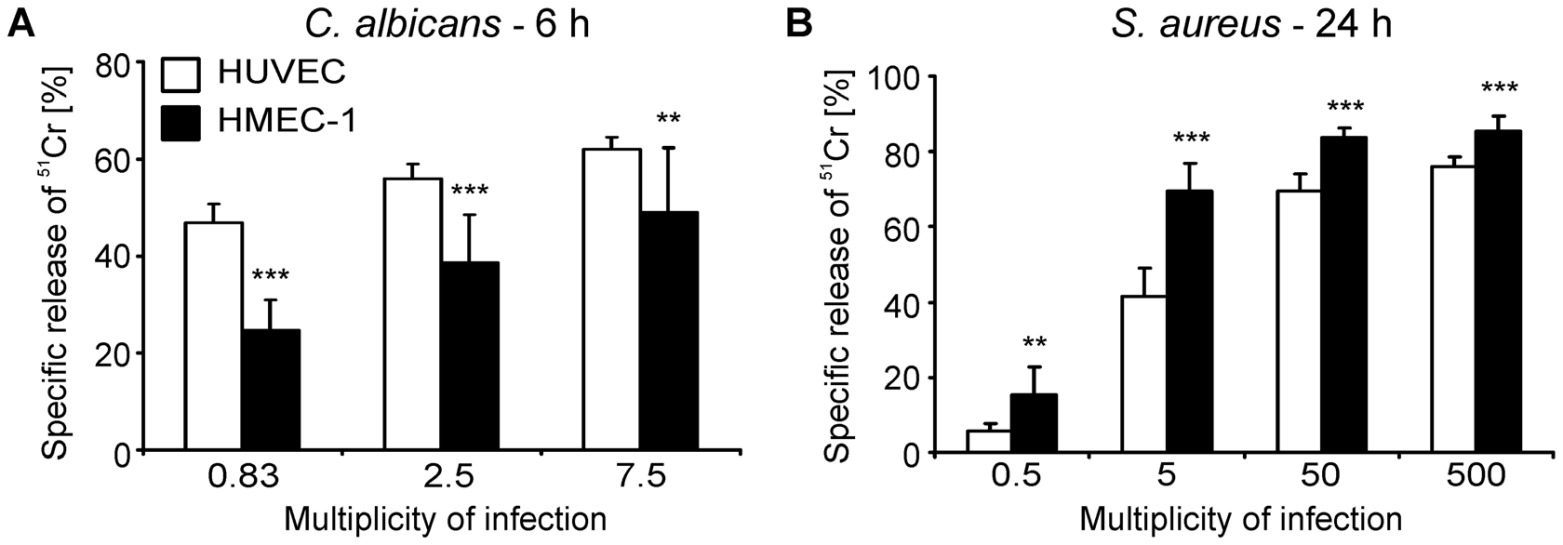
Microbial induced damage to HUVECs and HMEC-1 cells. The extent of damage to the indicated endothelial cells was determined by a ^51^Cr release assay, and was measured after 6 h of infection with *C. albicans* CAI4+CIp10 (A) and after 24 h of infection with *S. aureus* 6850 (B). **, *p*<0.01 and ***, *p*<0.001 in HMEC-1 vs. HUVECs.

The susceptibility of HMEC-1 cells and HUVECs to damage induced by *C. albicans* was determined by a ^51^Cr release assay. We found that HMEC-1 cells were significantly less susceptible than HUVECs to damage caused by the wild-type strain. For example, at the lowest multiplicity of infection (MOI), *C. albicans* induced 50% less damage to HMEC-1 cells as compared to HUVECs (**Fig. 2A**). Furthermore, both the *ssa1*Δ/Δ and *als3*Δ/Δ mutants caused significantly less damage to HMEC-1 cells than to HUVECs (**Table 3**). This reduction in damage was due to deletion of *ALS3* and *SSA1* because integrating an intact copy of these genes into the respective deletion mutants restored their capacity to damage both types of endothelial cells. Interestingly, both the *als3*Δ/Δ and *ssa1*Δ/Δ mutants had significantly greater damage defects on HMEC-1 cells than on HUVECs. Collectively, these results indicate that HMEC-1 cells are more resistant to *C. albicans*-induced damage than HUVECs. In addition, the presence of Ssa1 and Als3 on the surface of *C. albicans* is more important for damage of HMEC-1 cells than HUVECs.

**Table 3:** Capacity of different *C. albicans* and *S. aureus* mutants to damage HUVECs and HMEC-1 cells.

	HUVEC	HMEC-1	*p*
Strain	(% ± SD of the corresponding parental strain)		(HUVEC vs. HMEC-1)
*C. albicans* a			
*ssa1*Δ*/*Δ	74.7±9.4***	38.1±12.8***	<0.0001
*ssa1*Δ/Δ+*SSA1*	103.3±9.1	104.0±16.8	0.922
*als3*Δ*/*Δ	36.7±6.8***	5.8±5.1***	<0.0001
*als3*Δ*/*Δ+*ALS3*	101.7±3.7	100.1±5.2	0.448
*S. aureus* b			
6850 JB-1 (SCV)	5.2±4.6***	14.1±6.7***	<0.0001
300-169Δ*agr*	76.3±9.7***	26.6±3.7***	<0.0001

a , *C. albicans* induced damage was tested after 6 h at a MOI of 2.5.

b , *S. aureus* induced damage was tested after 24 h at a MOI of 50.

*** , *p*<0.001 vs. corresponding parental strain. Endothelial cell damage of parental strain was set to 100%.

In contrast to *C. albicans*, wild-type *S. aureus* caused significantly more damage to HMEC-1 cells than to HUVECs (**Fig. 2B**). As expected [14], the SCV and *agr* mutant strains had reduced capacity to damage both endothelial cell types (**Table 3**). However, the SCV strain caused significantly greater damage to HMEC-1 cells than to HUVECs, whereas the *agr* mutant caused greater damage to HUVECs than to HMEC-1 cells. Therefore, the mechanisms by which *S. aureus* damages HMEC-1 cells and HUVECs are likely to be different.

### HMEC-1 Cells Secreted Little IL-8 in Response to *C. albicans* and *S. aureus* Infection

**Figure 3:**
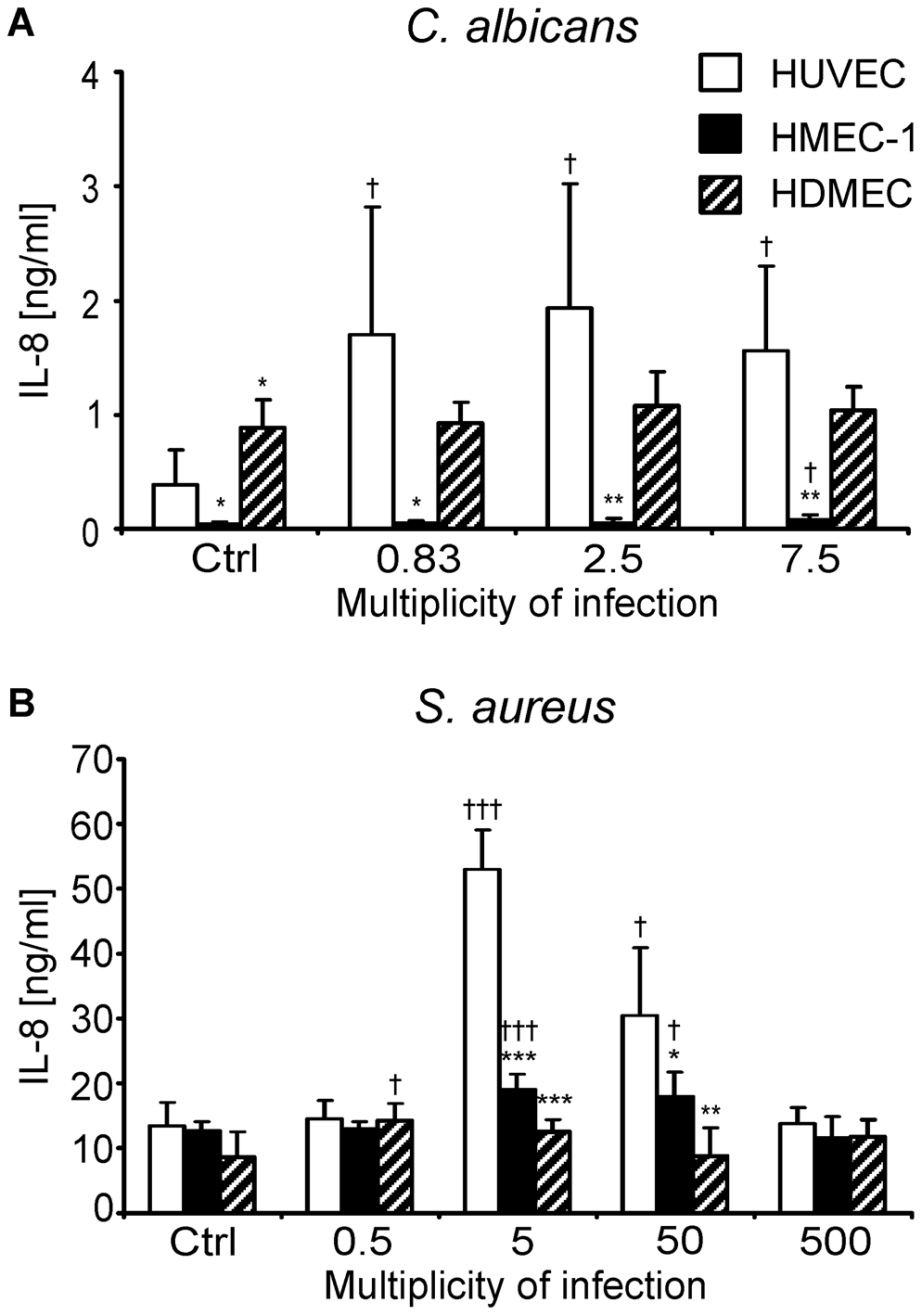
Endothelial cell stimulation. IL-8 levels in conditioned media of HUVECs, HMEC-1 cells, and HDMECs were determined 6 h after infection with *C. albicans* DAY185 (A) and 24 h after infection with *S. aureus* 6850 (B). Ctrl, uninfected controls; *, *p*<0.05; **, *p*<0.01; and ***, *p*<0.001 in HMEC-1 or HDMEC vs. HUVECs; ^†^, *p*<0.05; and ^†††^, *p*<0.001 in conditioned media vs. uninfected controls.

The proinflammatory chemokine, IL-8, plays a key role in activating and recruiting neutrophils and monocytes to sites of infection [36]. We therefore compared the secretion of IL-8 by HMEC-1 cells vs. HUVECs in response to microbial infection. After 6 h, uninfected HMEC-1 cells secreted approximately 10-fold less IL-8 than did HUVECs (**Fig. 3A**). Furthermore, when HMEC-1 cells were infected with wild-type *C. albicans*, secretion of IL-8 increased by a maximum of only 1.8-fold (*p* = 0.138). In contrast, infection of HUVECs with wild-type *C. albicans* resulted in up to a 5-fold increase in IL-8 secretion, depending on the MOI (*p*<0.05 for all MOIs tested). Thus, IL-8 secretion by HMEC-1 cells is poorly responsive to *C. albicans* infection.

HMEC-1 cells also responded weakly to *S. aureus*. During the 24 h incubation period, the basal release of IL-8 in the absence of infection was similar for HMEC-1 cells and HUVECs (**Fig. 3B**). Infection of HMEC-1 cells with *S. aureus* stimulated only a slight increase in IL-8 secretion at all MOIs tested. For instance, maximal increase in IL-8 secretion occurred at an MOI of 5, which resulted in only a 1.5-fold induction compared to uninfected control HMEC-1. In contrast, IL-8 production by HUVECs was highly inoculum-dependent. While the lowest inoculum tested (MOI of 0.5) did not induce a significant increase in IL-8 secretion, infection at MOIs of 5 and 50 led to a 4-fold and 2.3-fold increase in IL-8 secretion, respectively. At the highest MOI tested (500) there was no detectable increase in IL-8 secretion, probably because most endothelial cells were killed by this high inoculum. Collectively, these results indicate that HMEC-1 cells produce very little IL-8 in response to infection with either *C. albicans* or *S. aureus*, whereas IL-8 secretion by HUVECs is strongly induced by both organisms.

To investigate whether the low IL-8 production by the HMEC-1 cells was due to the anatomic source of these endothelial cells or their viral transformation, we analyzed the IL-8 response of primary human dermal microvascular endothelial cells (HDMECs). The basal release of IL-8 by these endothelial cells was significantly greater than that of both HUVECs and HMEC-1 cells after 6 h (**Fig. 3**). However, infection with three different inocula of *C. albicans* did not induce a significant increase in IL-8 production by HDMECs (**Fig. 3A**), and *S. aureus* induced only a 1.6-fold increase in IL-8 production at an MOI of 0.5 (**Fig. 3B**). Therefore, although the low basal production of IL-8 by HMEC-1 cells is likely the result of their transformation, the minimal increase in IL-8 production in response to *C. albicans* and *S. aureus* infection is probably due to the anatomic source of these endothelial cells.

## Discussion

Although both HUVECs and HMEC-1 cells have been used to investigate the interactions of microbial pathogens with endothelial cells *in vitro*, there have been relatively few studies that directly compared the response of these two types of endothelial cells to infection by prototypical endovascular pathogens. We determined that *C. albicans* and *S. aureus* interacted significantly differently with HMEC-1 cells as compared to HUVECs. For example, wild-type *C. albicans* was less adherent to HMEC-1 cells than to HUVECs. Furthermore, although we found that the *als3*Δ/Δ and *ssa1*Δ/Δ mutants had reduced adherence to both types of endothelial cells when tested under static conditions, it remains possible that *C. albicans* adheres by different mechanisms to HMEC-1 cells than to HUVECs under flow conditions. For instance, one group reported that *C. albicans* hyphae are less adherent than yeast to HMEC-1 cells under flow conditions [29], whereas another group found that *C. albicans* hyphae have greater adherence to HUVECs than do yeast-phase organisms under conditions of flow [37]. Collectively, these results suggest that the endothelial cells receptors that are bound by *C. albicans* may differ in either their expression level or composition between HMEC-1 cells and HUVECs.

We also found that *C. albicans* hyphae were endocytosed less avidly by HMEC-1 cells than by HUVECs. It is known that *C. albicans* hyphae induce their own endocytosis by binding to N-cadherin on the surface of endothelial cells [38]. Although both HUVECs and HMEC-1 cells express N-cadherin [38], it is possible that the binding of *C. albicans* to N-cadherin may activate divergent signaling pathways in these two types of cells. Alternatively, receptors other than N-cadherin, which also mediate the endocytosis of *C. albicans* might be expressed at higher level by HUVECs than by HMEC-1 cells.

In addition, we determined that wild-type *S. aureus* cells were endocytosed similarly by HUVECs and HMEC-1 cells, whereas an SCV strain had markedly enhanced invasion of both endothelial cell types as compared to its parental isolate. SCV strains have previously been reported to have increased invasion of HUVECs [14], [34] and bovine aortic endothelial cells [39], probably due to enhanced expression of fibronectin-binding proteins, which are the essential mediators of *S. aureus* invasion of host cells [40]–[42].

Interestingly, although an *agr* mutant had normal adherence to both endothelial cell types as compared to its parental strain, it was defective in invading HMEC-1 cells, but not HUVECs. The role of *agr* in governing *S. aureus* adherence to endothelial cells is known to be influenced by multiple factors, including the strain background, growth phase, and whether adherence is measured under static or flow conditions [43], [44]. Thus, even though *agr* did not affect endothelial cell adherence in our investigations, it might have done so under different experimental conditions. In concordance with our data, others have found that *agr* is dispensable for *S. aureus* invasion of HUVECs [32]. However, our finding that *agr* is necessary for maximal invasion of HMEC-1 cells again demonstrates that *S. aureus* interacts differently with this endothelial cell line as compared to HUVECs.

As expected, *C. albicans* caused significant damage to HMEC-1 cells and HUVECs. However, we found that wild-type *C. albicans* caused significantly less damage to HMEC-1 cells than to HUVECs. Furthermore, damage to HMEC-1 cells was more dependent on the presence of the *C. albicans* Ssa1 and Als3 invasins than was damage to HUVECs. It is known that *C. albicans* must invade endothelial cells to cause maximal damage to these cells [6]. However the *ssa1*Δ/Δ and *als3*Δ/Δ mutant strains had similar defects in their capacity to invade both HMEC-1 cells and HUVECs. Thus, the differential susceptibility of HMEC-1 cells vs. HUVECs to damage caused *C. albicans* might be due to differences in post-invasion processes such as intracellular trafficking or activation of signal transduction pathways.

In sharp contrast to *C. albicans*, *S. aureus* caused significantly more damage to HMEC-1 cells as compared to HUVECs. Because wild-type *S. aureus* cells were endocytosed similarly by both types of endothelial cells, the differential susceptibility of HMEC-1 cells and HUVECs to *S. aureus*-induced damage must also be due to post- endothelial cell invasion events. Importantly, the hyper-adherent SCV strain had the greatest damage defect on HUVECs, whereas the *agr* mutant had the greatest damage defect on HMEC-1 cells. Thus, the changes in *S. aureus* that occur during SCV formation influence its ability to damage HUVECs more than HMEC-1 cells. Conversely, factors controlled by *agr* (e.g., cytolytic toxins) mediate *S. aureus*-induced damage of HMEC-1 cells more than HUVECs.

We found that both *C. albicans* and *S. aureus* induced HUVECs to secrete IL-8, as has been reported previously [8], [45]–[47]. However, under the conditions tested, *C. albicans* did not stimulate significant IL-8 production by HMEC-1 cells, while *S. aureus* induced only a minor increase in IL-8 production by these endothelial cells. Of note, HMEC-1 cells have been shown to produce much lower levels of IL-8 than do HUVECs in response to other stimuli, such as IL-1β and *Brucella abortus* [23], [48]. Importantly, the production of additional proinflammatory and proangiogenic factors such as IL-6, vascular cell adhesion molecule-1, E-selectin, and vascular endothelial cell growth factor is much lower in HMEC-1 cells as compared to HUVECs [17], [18]. Our data with primary HDMECs suggest that while the low basal production of IL-8 by the HMEC-1 cells may be due to their transformation, the poor IL-8 production by these endothelial cells in response to *C. albicans* and *S. aureus* may be a general property of dermal microvascular endothelial cells. Nevertheless, these collective findings suggest that HUVECs are preferable to HMEC-1 cells for studying the effects of *C. albicans* and *S. aureus* on stimulation of the endothelial cell proinflammatory response.

In summary, our current data indicate that *C. albicans* and *S. aureus* interact with HMEC-1 cells significantly differently than with HUVECs. Thus, data obtained with one type of endothelial cell cannot necessarily be extrapolated to other types obtained from different vascular beds. Furthermore, both primary and transformed dermal microvascular endothelial cells release very little IL-8 above basal levels in response to *C. albicans* or *S. aureus* infection, in contrast to HUVECs. On the other hand, HMEC-1 cells may be particularly useful for investigating mechanisms of endothelial cell damage that depend upon the *C. albicans* Ssa1 and Als3 invasins or the target genes of the *S. aureus agr* regulon. Moreover, a key consideration when deciding which type of endothelial cell to use to investigate microbial-endothelial cell interactions *in vitro* is how well these interactions are predictive of the events that occur during *in vivo* infection. We have found previously that the capacity of different strains of *C. albicans* and *S. aureus* to damage HUVECs *in vitro* is directly correlated with their virulence in animal models of hematogenous infection [13], [14]. Whether one or more interactions of these microbial pathogens with HMEC-1 cells is also predictive of virulence will be the focus of future investigations.

## Materials and Methods

### Ethics Statement

The protocol for collecting umbilical cords for the harvesting of HUVECs used in these studies was approved by the Institutional Review Board of the Los Angeles Biomedical Research Institute at Harbor-UCLA Medical Center. This protocol was granted a waiver of consent because the donors remained anonymous.

### Endothelial Cell Culture

HUVECs were harvested from human umbilical cord veins by the method of Jaffe *et al*. [49], and maintained in complete M199 medium (with 10% fetal bovine serum and 10% bovine calf serum, plus penicillin, 100 IU/ml; streptomycin, 100 µg/ml) as previously described [6]. HUVECs were routinely used at passage 3 for various assays. HMEC-1 cells were obtained from Kathryn Kellar of the Centers for Disease Control (CDC), and maintained as recommended [21]. HDMECs were purchased from Lonza BioResearch and grown as directed. All experiments in which HUVECs were compared with HMEC-1 cells were performed in parallel.

### Bacterial and Fungal Strains and Growth Conditions

We selected *C. albicans* and *S. aureus* for these investigations as they represent two prototypical endovascular pathogens. All *C. albicans* and *S. aureus* strains used in this study are listed in **Table 1**. For use in the experiments, *C. albicans* yeast-phase cells were grown in YPD (1% yeast extract, 2% Bacto-peptone, 2% D-glucose) broth overnight in a rotary shaker at 30°C. All *S. aureus* strains were grown overnight in Bacto BHI broth (Difco) without shaking at 37°C. On the day of the experiment, the organisms were harvested by centrifugation and washed with PBS. The *C. albicans* cells were counted using a hemacytometer and the *S. aureus* cells were adjusted to an OD_600_ of 0.5 and diluted accordingly. *S. aureus* inocula were confirmed by quantitative culture. The construction of the *C. albicans ssa1*Δ/Δ+*SSA1* and *als3*Δ/Δ+*ALS3* complemented strains was described previously [12], [15]. Attempts to genotypically and phenotypically complement the entire *agr* operon in the 300-169Δ*agr* mutant were unsuccessful, as has been reported by others [50].

### Adherence and Endocytosis Assays

The capacity of the various *C. albicans* strains to adhere to and be endocytosed by HUVECs and HMEC-1 cells was quantified using our previously described differential fluorescence assay [13]. Briefly, the endothelial cells were grown on 12-mm-diameter glass cover slips and inoculated at a MOI of 1 in RPMI 1640 medium. After 1.5 h, the nonadherent organisms were removed by rinsing with Hanks balanced solution (HBSS), after which the cells were fixed with 3% paraformaldehyde. The adherent, extracellular organisms were stained with anti-*C. albicans* rabbit antiserum (Biodesign International) conjugated with Alexa Fluor 568 (red fluorescence; Molecular Probes). Next, the host cells were permeabilized in 0.5% Triton X-100, after which the cell-associated organisms (the adherent plus endocytosed organisms) were stained with anti-*C. albicans* antiserum conjugated with Alexa Fluor 488 (green fluorescence). For ease of discussion, organisms that were host-cell associated are referred to as “adherent”. The cover slips were mounted inverted on a microscope slide, and the number of endocytosed and cell-associated organisms was determined by viewing the cells with an epifluorescent microscope. At least 100 organisms were counted on each cover slip, and organisms that were partially internalized were scored as being endocytosed. Each experiment was carried out in triplicate on at least three separate occasions.

The capacity of the various *S. aureus* strains to adhere to and be endocytosed by endothelial cells was quantified using the lysostaphin protection assay [51], [52]. Briefly, endothelial cells were grown to confluency and inoculated at a MOI of 1 in M199 invasion medium (M199 without antibiotics and serum, but with 1% human albumin, [14], [32]). After 3 h, one half of wells were washed three times with HBSS, after which the endothelial cells were detached and then lysed. The number of host-cell associated *S. aureus* was determined by quantitative culture. To determine the number of internalized bacteria, complete M199 medium with 10 µg/ml lysostaphin was added to the endothelial cells. After 20 min incubation, the wells were rinsed three times and the number of internalized bacteria was determined by lysing the host cells with cold distilled water followed by quantitative culture. Each strain was tested in duplicate in at least three different experiments.

### Endothelial Cell Damage Assay

The extent of endothelial cell damage caused by the different strains of *C. albicans* was measured using our previously described ^51^Cr release assay [6]. Briefly, host cells were loaded with 6 µCi/ml Na_2_^51^CrO_4_ (MP Biomedicals) overnight. After removing the unincorporated ^51^Cr by rinsing, the cells were infected with yeast of the various *C. albicans* strains resuspended in RPMI 1640. The infected host cells were incubated for 6 h, after which the amount of ^51^Cr released into the medium and retained by the cells was determined by γ-counting. Wells containing host cells, but no organisms, were processed in parallel to determine the spontaneous release of ^51^Cr.

The amount of endothelial cell damage induced by *S. aureus* was also determined with a ^51^Cr release assay as previously described [14]. Briefly, bacteria were added to the endothelial cells in M199 invasion medium for 3 h, after which 500 µl of fresh complete M-199 medium containing 10 µg/ml lysostaphin was added [51], [53]. Damage was determined 24 h after infection at the indicated MOIs. Each strain and condition was tested in triplicate on at least three different occasions.

The optimal time for damage assay assessments for the two different organisms was determined after extensive pilot studies.

### Detection of IL-8 in Conditioned Media

The effects of *C. albicans* and *S. aureus* on endothelial cell secretion of IL-8 were determined using the same inocula and incubation conditions as in the damage experiments, except that M-199 medium was used in both the *C. albicans* and *S. aureus* experiments. At the end of the defined incubation period, the conditioned medium above the endothelial cells was collected, centrifuged at 1,000 g to pellet the cells, and then frozen in aliquots at −80C for later analysis. The concentrations of IL-8 in conditioned media were determined by commercial enzyme-linked immunosorbent assays (Invitrogen) according to the manufacturer’s instructions. Each strain and condition was tested in duplicate in three independent experiments.

### Statistical Analyses

The data were analyzed using the two-tailed student’s t-test, and *p*-values ≤0.05 were considered statistically significant.
